# On the identity of the type species of *Parasa* (Lepidoptera: Limacodidae): investigations into the Nearctic *Parasa chloris* and related taxa

**DOI:** 10.1093/aesa/saaf016

**Published:** 2025-07-14

**Authors:** Tabitha R Taberer

**Affiliations:** Department of Biology, University of Oxford, Mansfield Road, Oxford OX1 3SZ, UK; African Natural History Research Trust, Street Court, Kingsland, Leominster HR6 9QA, UK

**Keywords:** taxonomy, type material, DNA barcoding, historic DNA, lectotype

## Abstract

The pantropical Limacodid genus *Parasa* Moore [1860] comprises a charismatic group of moths, whose adults display green banding on the forewing while the larvae are often brightly colored, possessing stinging hairs. Three previously unidentified syntypes of the type species *Parasa chloris* (Herrich-Schäffer [1854]) were identified in the National Museum of Natural History, Smithsonian Institution, USA, having passed through several collections over the past ca. 180 years. Described from specimens with a vague provenance, the true type locality was unveiled utilizing COI barcoding of the lectotype designated herein, together with other barcoded specimens from North and Central America, morphological observations in adults and male genitalia, as well as distribution records from museum specimens and the citizen science database iNaturalist. Results suggest the type locality of *P. chloris* as north-eastern USA, likely from the southern states. In addition, the nomenclatural history of *P. chloris* is here discussed in detail, and its synonyms are clarified with regard the morphologically-similar, sympatric species *Parasa indetermina* (Griffith and Pidgeon, 1832 *nec* Boisduval), and *Limacodes viridus* Reakirt (1864) **syn. rev.** is here revived as a synonym of the latter. Taxonomic remarks are also made regarding species closely related to *P. chloris* (*Parasa minima* (Schaus, 1892), *Parasa huachuca* Dyar (1905) **stat. nov.**, *Parasa cuernavaca* Dyar (1907) **stat. rev.**, and *Parasa maysi* Schaus (1920)), resulting from COI barcoding, and morphological examinations of all primary type and additional material. This research represents the first step in delimiting *Parasa* in preparation for future taxonomic work testing the monophyly of this widespread genus.

## Introduction

*Parasa* Moore [1860] is a pantropical genus of slug moth in the family Limacodidae, characterized by typical green and brown patterning of the forewing, often with yellow or beige hindwings. The genus has previously received attention due to its unusually widespread distribution and superficially uniform phenotype across its range, although much confusion exists surrounding its taxonomic classification (Holloway 1986).

Its type species, *Neaera chloris*Herrich-Schäffer [1854], a purportedly widespread species throughout North America, has a convoluted nomenclatural and taxonomic history due in part to the vague type locality pointing to its origin in South America and the fact that the type material of Herrich-Schäffer’s taxon never having been traced. To complicate matters further, a phenotypically similar species, *Parasa indetermina* (Griffith and Pidgeon, 1832*nec* Boisduval) (the authorship of this species is discussed extensively below) exists in sympatry throughout eastern USA which has confounded many early authors, despite the greatly differing larvae of the 2 species being known about since the 19th century.

Authors generally agree that the American species may not be congeneric with African and Asian taxa, and that the monophyly of *Parasa* requires investigation (e.g., Epstein and Corrales 2004, Solovyev 2014, Lin et al. 2019). However, without establishing what “true” *Parasa* is, such work cannot be undertaken. In this present article, critical examinations of historical literature, exhaustive searches in museum collections, together with the synthesis of both morphological and barcode data from modern and old specimens, has enabled the taxonomy of *Parasa chloris* to be stabilized. The steps taken to reach this conclusion are outlined in detail below.

## Materials and Methods

### Morphological Investigations

Images of adults were taken using a Canon EOS 650d DSLR camera equipped with a Sigma DG Macro 105 mm 1:2.8 lens, or with a Canon EOS 5Ds with an MP-E 65 mm lens on a stacking setup. The genitalia were dissected and stained with Eosin Y, applying standard methods of preparation (Lafontaine and Mikkola 1987), then embedded in Euparal on microscope slides. The genitalia preparations were photographed using a Canon EOS 700D camera mounted on a Leitz Diaplan compound microscope. Terminology of genitalia follows Solovyev (2014).

Label data in quotation marks are transcribed verbatim, a new line denoted with “|” and a different label with “||.” Additional material examined can be found in Supplementary Information S1.

The majority of the point data for the distribution map was obtained from imaged iNaturalist “Research grade” records (www.inaturalist.org). Point data coordinates and sources can be found in Supplementary Information S2.

Museum abbreviations used throughout:

ANSP—Academy of Natural Sciences Philadelphia, Philadelphia, PA, USA

CMNH—Carnegie Museum of Natural History, Pittsburgh, PA, USA

FMNH—Field Museum of Natural History, Chicago, IL, USA

MCZ—Museum of Comparative Zoology, Harvard, Cambridge, MA, USA

MfN—Museum für Naturkunde, Berlin, Germany

MSU/ARC—Michigan State University, A.J. Cook Arthropod Research Collection, East Lansing, MI, USA

NHMUK—Natural History Museum, London, UK

NMUFRJ—Museu Nacional (National Museum of the Federal University of Rio de Janeiro), Rio de Janeiro, Brazil

PEM—Peabody Essex Museum, Salem, MA, USA

USNM—National Museum of Natural History, Smithsonian Institution, Washington D.C., USA

ZMH—Zoologisches Museum Hamburg, Hamburg, Germany

ZSM—Zoologische Staatssammlung München, Munich, Germany.

This article and the nomenclatural act(s) it contains have been registered in Zoobank (www.zoobank.org), the official register of the International Commission on Zoological Nomenclature. The LSID (Life Science Identifier) number of the publication is: urn:lsid:zoobank.org:pub:1BE40E3D-A878-47D3-BEA0-23FB453F9EBF.

### Genetic Investigations

Abdominal tissue of 2 putative type specimens (USNMENT01848067 and USNMENT01848068) was submitted to the Canadian Centre for DNA Barcoding (CCDB, Biodiversity Institute of Ontario, University of Guelph) for extraction, amplification, and sequencing of cytochrome oxidase subunit I (COI-5P). Methodology followed those described in D’Ercole et al. (2021) and Prosser et al. (2016), developed for highly degraded museum samples whereby multiple short-length (~150 bp) amplicons were used to assemble the COI barcode as a result of multiplex PCR. Despite the age of the specimens (~180 years), the methodology implemented at the CCDB successfully generated sequence fragments of varying length from both type specimens. However, due to high levels of fragmentation, only short fragments could be recovered and these could not be assembled into a longer COI contig. Nevertheless, these fragments were then compared via BLAST to all available unique BOLD BINs to assign taxonomy.

Sequences assigned by BLAST as any taxa other than *Parasa chloris* were removed from the dataset, leaving zero sequences for specimen USNMENT01848067 and 8 sequences for USNMENT01848068. As an additional filter due to requiring a close match to modern *P. chloris* sequences, a threshold value of > 97% percentage identity and 1e-50 was implemented on the BLAST search results, resulting in retention of one matching sequence (Table 1).

**Table 1.** The sequence obtained from *Parasa chloris* type specimen USNMENT01848068 retained after a BLAST search of all available BOLD BINs, filtering of *Parasa chloris* identified samples and implementation of a percentage identity threshold > 97% and an e-value threshold of 1e-50. The reference sample within the given closest matching BOLD BIN is provided as per BLAST search results

Sequence ID	e-value	Percentage identity (%)	Coverage (bp)	BIN	Reference sample in BIN
*Parasa chloris* type PCTSI002-24	8e^−79^	97.04	169	BOLD:AAD4294	LGSM135-04

The type sequence was then aligned to 27 modern COI sequences currently classified as *P. chloris* and *P. minima*Schaus (1892) as obtained from the public database on BOLD using MUSCLE in Aliview v1.28. Two *P. indetermina* and 2 *P. viridogrisea* (Dyar 1898a) specimens were included as outgroup taxa to root the phylogeny. The resulting alignment was manually examined for evidence of contamination events or chimeras resulting from the inclusion of non-target sequences in the assembly. As several fragments, many of them very short, were present in the type specimen alignment, only the longest fragment was retained and aligned to the modern specimens. The resulting complete alignment was 658 bp, with the type sequence having a single continuous sequence of 177 bp. Although comparably short to the COI barcode, even short sequences have can provide valuable taxonomic information (Meusnier et al. 2008), and reliable connections to full-length barcodes can be made to individuals of the same species (Hebert et al. 2013).

Maximum likelihood phylogenetic tree searches were performed via RAxML (Stamatakis 2014) with 1000 bootstrap replicates and a GTR + FO model. Pairwise distances within and between species were calculated using the Kimura 2-parameter model (Kimura 1980) in MEGA v.11.0.13. The resulting phylogeny was visualized and annotated in FigTree v1.4.4 and Abode Photoshop v.25.12.0 (Fig. 3).

## Results

### Description and Designation of the Type Species of *Parasa*

**Fig. 1.**
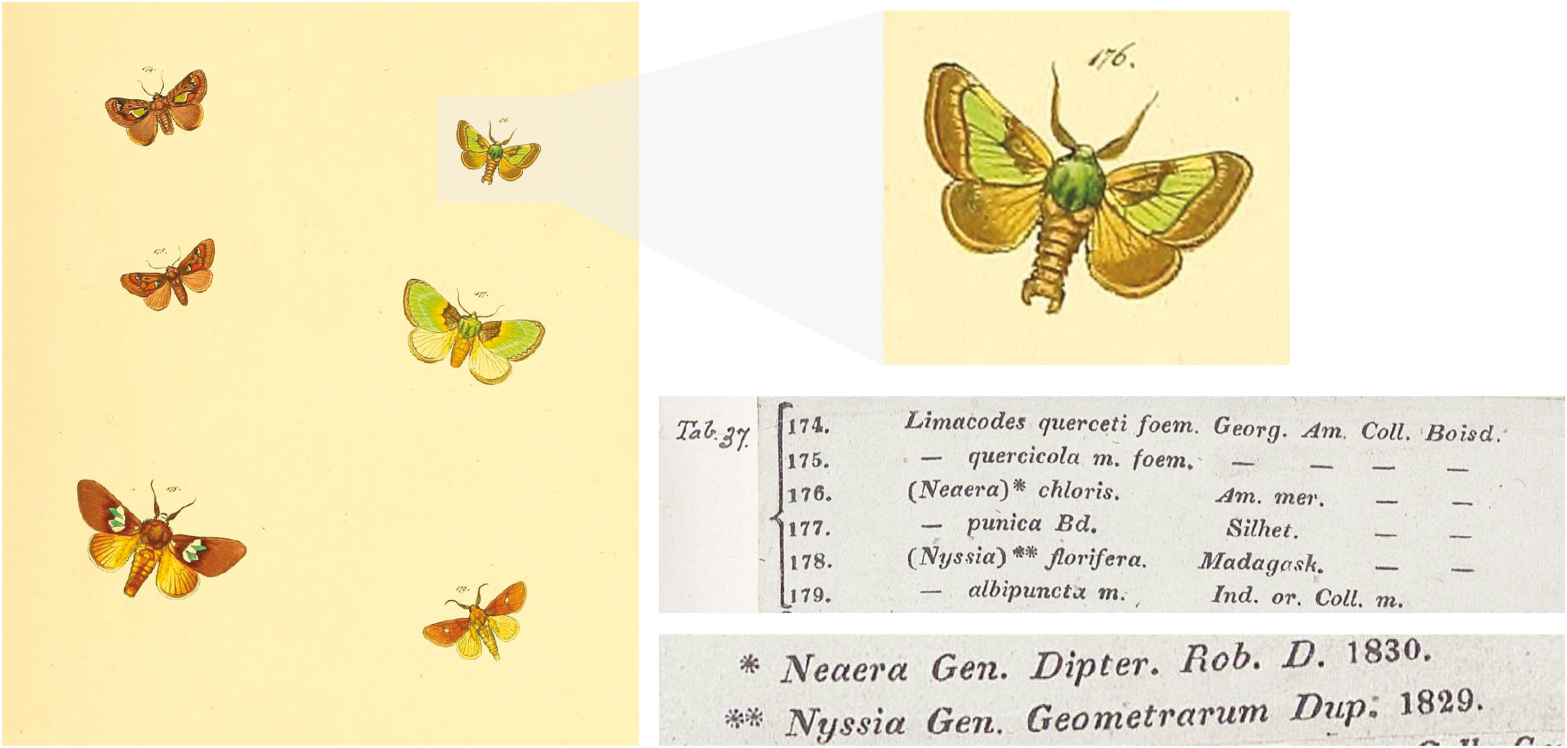
Complete original description of *Parasa chloris* (Herrich-Schäffer [1854]) in *auss. Schmett.*, consisting of the colored illustration (denoted as figure 176 on plate 37) and the plate wrapper (scan courtesy of the NHMUK). The species was originally described within the genus *Neaera*Herrich-Schäffer [1854], although it was realized that this was a junior homonym of the Diptera genus *Neaera*Robineau-Desvoidy (1830) (denoted with *). The right-hand column shows that *P. chloris* was described from specimen(s) belonging to the collection of Boisduval.

**Fig. 2.**
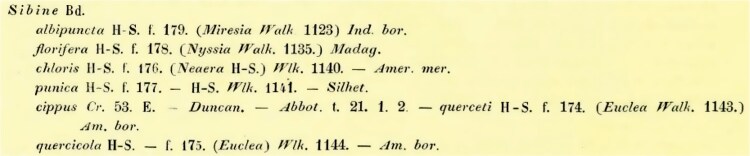
Text portion of *auss. Schmett.* (Herrich-Schäffer [1858]), published 4 years after the original description of *Parasa chloris*. Here, *P. chloris* was transferred to *Sibine*Herrich-Schäffer (1855). The “f. 176” refers to figure 176 of the original illustration of Herrich-Schäffer ([1854]) (as shown here in Fig. 1), and “Wlk. 1140” denotes the written description of *P. chloris* on page 1140 of Walker (1855).

*Neaera chloris* was described by Herrich-Schäffer (1799 to 1874) in *Sammlung neuer oder wenig bekannter aussereuropäischer Schmetterlinge* (herein referred to as *auss. Schmett.*), as a species in his new genus *Neaera*Herrich-Schäffer [1854]. The description appeared as an illustration on a plate published in 1854, which was issued with a wrapper containing information on the species (Fig. 1). Before publication, Herrich-Schäffer ([1854]) had realized that *Neaera* was a junior homonym of the genus *Neaera*Robineau-Desvoidy (1830) (Diptera: Tachinidae) (as shown on the plate wrapper; Fig. 1) and thus transferred *chloris* (and several limacodid species) into the genus *Sibine*Herrich-Schäffer [1855] (Lepidoptera: Limacodidae) in the text portion of *auss. Schmett.* (Herrich-Schäffer [1858]) (Fig. 2). Moore ([1860]) proposed the name *Parasa* to replace *Neaera*, and although *N. chloris* was not specifically dealt with, it was implicitly transferred to *Parasa* together with all of Walker’s (1855) Asian *Neaera* species. The first reference to *Parasa chloris* in the literature was made by Walker (1862), followed several years later by Grote and Robinson (1868a). The confusion regarding the early nomenclature of the type species is ultimately rooted in the pantropical nature of the genus, compounded by various taxonomists working on the fauna of different regions. *Neaera chloris* was subsequently designated as the type species of *Parasa* by Fletcher and Nye (1982).

**Fig. 3.**
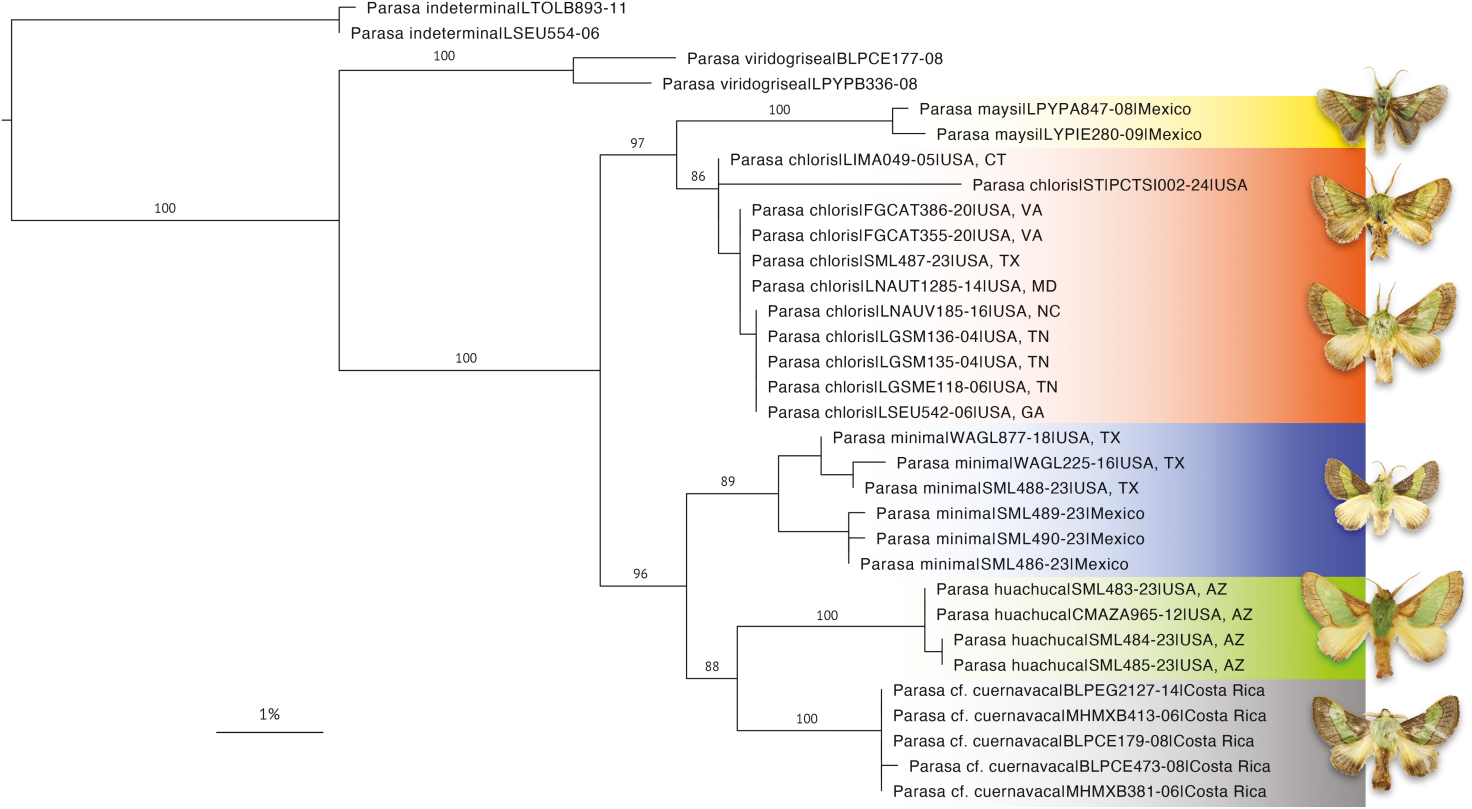
Maximum likelihood phylogeny of *Parasa chloris* and related species, including a ~180-year-old syntype specimen (PCTSI002-24). Bootstrap values are provided above the branches. Scale bar represents substitution rate per site.

### Locating the Type Specimen of *Parasa chloris*

**Fig. 4.**
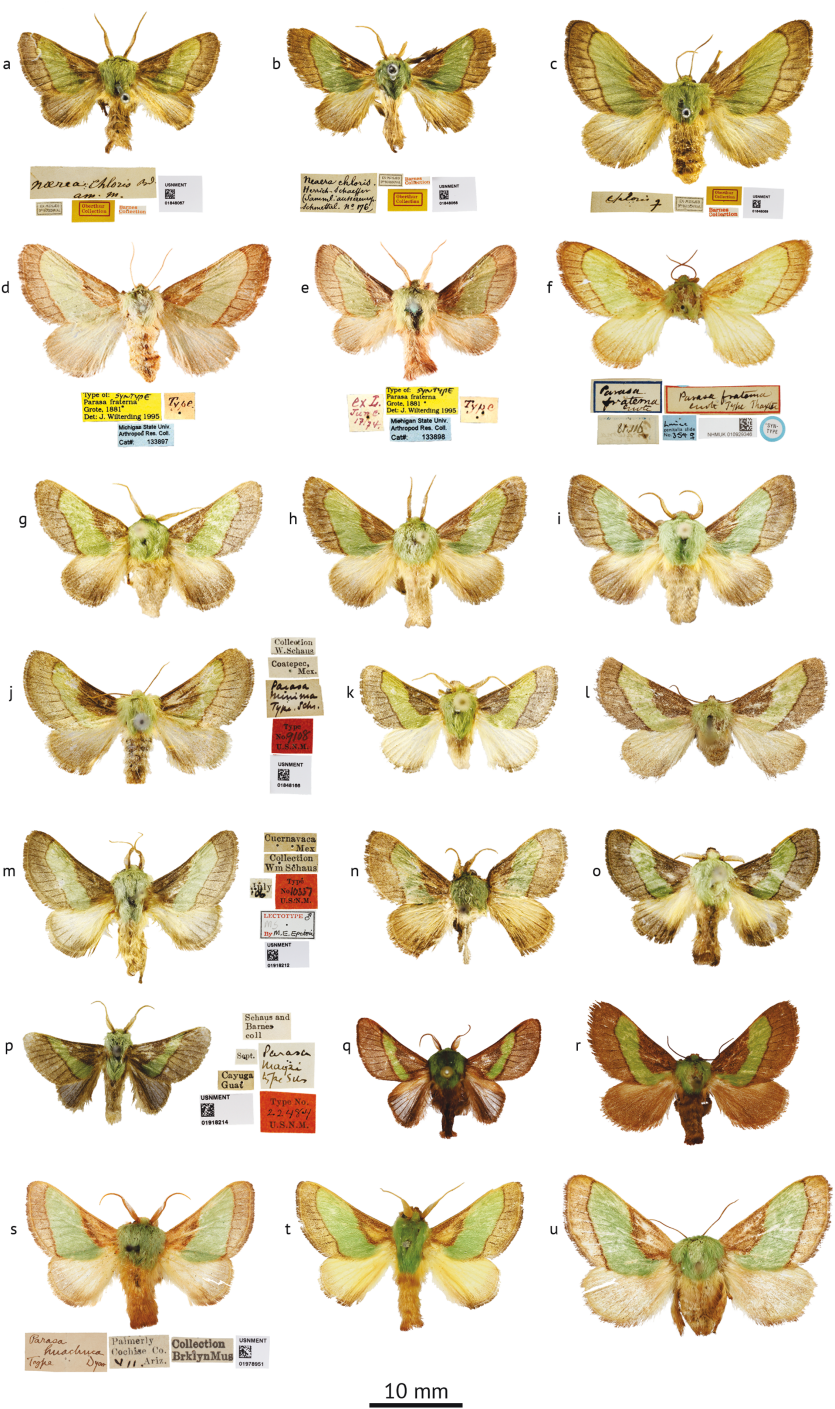
*Parasa* species, adults. Figures a-i: *Parasa chloris* (Herrich-Schäffer). a. Lectotype ♂ of *Neaera chloris* Herrich-Schäffer; b. Paralectotype ♂ of *Neaera chloris*; c. Paralectotype ♀ of *Neaera chloris* (USNM); d. Syntype ♀ of *Parasa fraterna* Grote; e. Syntype ♂ of *Parasa fraterna* (MSU/ARC); f. Syntype ♀ of *Parasa fraterna* (NHMUK); g. ♂, Bossier Parish, Louisiana, USA, USNMENT01918180; h. ♂, Virginia, Fairfax County, USA, USNMENT01918176; i. ♂, Ohio, Summit County, USA, USNMENT01918175 (USNM); Figures j-l: *Parasa minima* Schaus. j. Holotype ♀; k. ♂, Texas, Hidalgo County, USA, USNMENT01848905; l. ♀, Texas, Hidalgo County, USA, USNMENT01978145 (USNM); Figures m-n: *Parasa cuernavaca* Dyar. m. Syntype ♂ of *Parasa chloris* var. *cuernavaca* Dyar; n. ♂, Sinaloa, Mexico, USNMENT01848165; Figure o: *Parasa* cf. *cuernavaca*, ♂, Área de Conservación Guanacaste, Costa Rica, USNMENT01918183 (USNM); Figures p-r: *Parasa maysi* Schaus. p. Holotype ♂ (USNM); q. ♂, Las Cuevas, Belize; r. ♀, Cayo, Belize (NHMUK); Figures s-u: *Parasa huachuca* Dyar. s. Holotype ♂ of *Parasa chloris* var. *huachuca* Dyar (USNM); t. ♂, Arizona, Madera Canyon, USA, NHMUK010929984 (NHMUK); u. ♀, Arizona, Miller Canyon, USA, USNMENT01978147 (USNM).

The name-bearing type of *Parasa chloris* has previously never been traced, likely due to the vague original description provided by Herrich-Schäffer ([1854]). As the type specimen represents a fixed definition of a species (ICZN, Article 61), and the fact that *P. chloris* represents the type species of its genus, the absence of an accurate identification of *Parasa* could render further revisionary work as incorrect or incomplete. Efforts were here made to locate the type material of *P. chloris*, resulting in the identification of 3 syntypes (Fig. 4a-c) which had been passed through numerous collections and are currently housed in the USNM. In order to fix the identity of this taxon, and in accordance with ICZN Article 74.7 Recommendation 74B, specimen USNMENT01848067 (Fig. 4a) is here designated the lectotype of *Neaera chloris*. A detailed account of the efforts made to trace the type specimens can be found in Supplementary Information S3.

### Type Locality of *Parasa chloris*

**Fig. 5.**
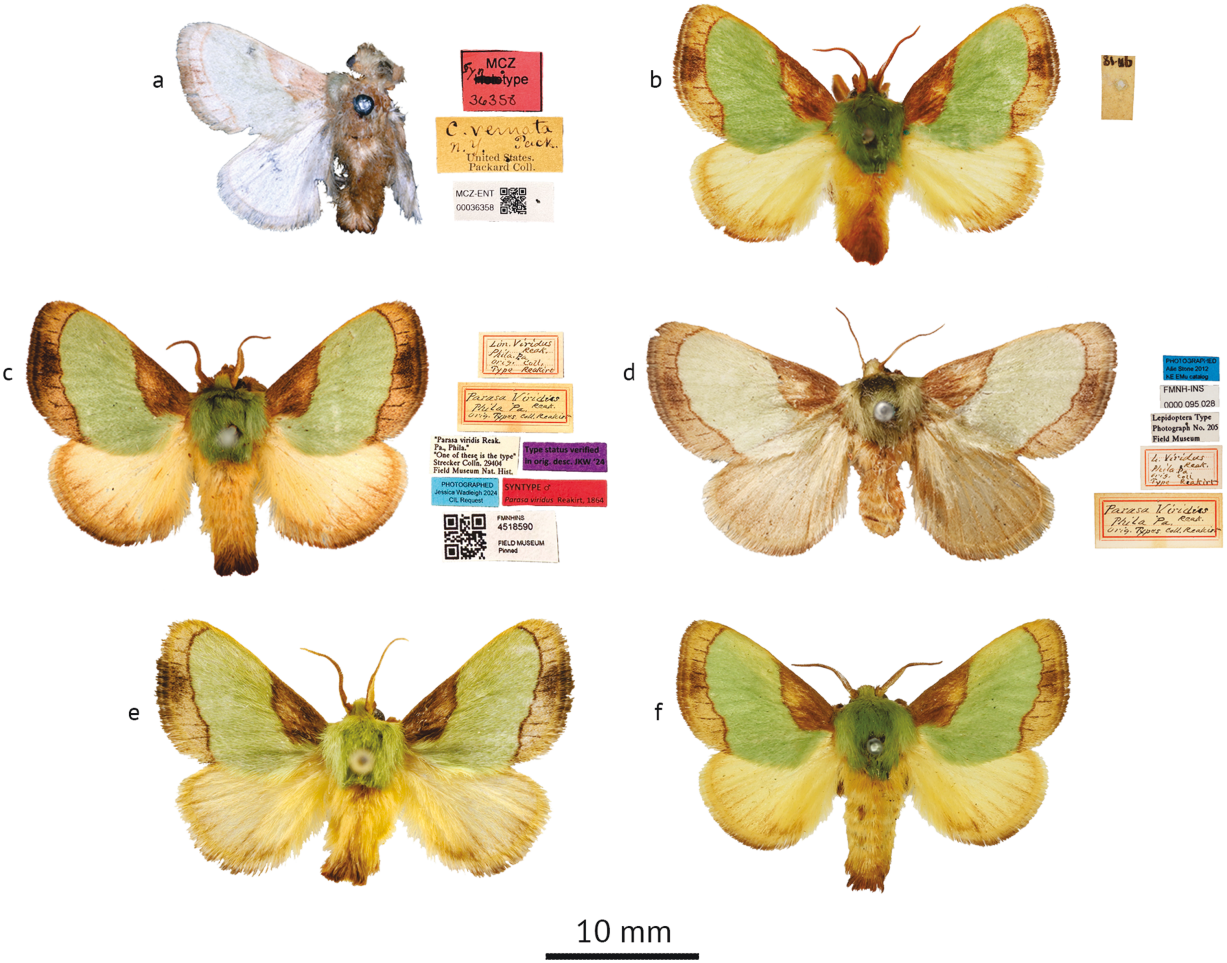
*Parasa indetermina* (Griffith & Pidgeon) adults. a. Possible syntype ♂ of *Callochlora vernata* Packard (MCZ); b. Possible syntype, ♂ of *Callochlora vernata* (NHMUK); c. Syntype ♂ of *Limacodes viridus* Reakirt; d. Syntype ♀ of *Limacodes viridus* (FMNH); e. ♂, Maryland, Patuxent Wildlife Research Center, USA, USNMENT01918182 (USNM); f. ♂, Texas, USA, NHMUK010929982 (NHMUK).

**Fig. 6.**
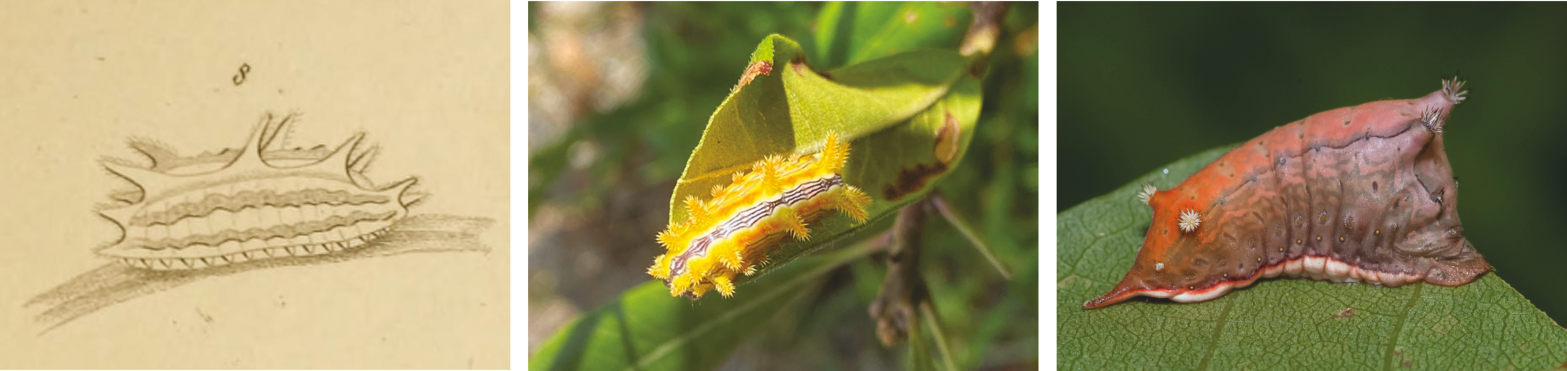
*Parasa* larvae. Left. Illustration of *P. indetermina* in Guérin-Méneville (1832); Middle. *P. indetermina* larva feeding on Myrica, Cape May, NJ (photo: Tabitha R. Taberer); Right. *Parasa chloris* larva (photo: Bonnie Ott; https://www.flickr.com/people/sparrowbon/).

The vague locality data associated with the *Parasa chloris* lectotype label as shown in Fig. 4a states “*am. m*.” which is slightly extended to “*Amer. mer*.” in Herrich-Schäffer ([1858]) seemingly referring to “South America” (“America meridionalis” in Latin or “Amérique méridionale” in French). However, *P. chloris* is typically cited as a North American species, particularly from the east coast of the United States, and is included as such in recent publications (e.g., Epstein and Fiesler 2023). This is in part due to its association with another phenotypically similar species, *Parasa indetermina* (found from north-eastern USA) through the early literature, whose external morphology (Fig. 5) shares similarities to *P. chloris* despite the larvae being largely different (Fig. 6), as well as 3 junior synonyms (*Callochlora vernata*Packard (1864), Fig. 5a-b, *Limacodes viridus*Reakirt (1864), Fig. 5c-d, and *Parasa fraterna*Grote (1881), Fig. 4d-f) which have been repeatedly swapped between the 2 species during the 19th century. These relationships are resolved fully below. A full account of how *P. chloris* has been treated throughout the literature is provided in Supplementary Information S4.

In the absence of further literature-based information regarding the origin of Boisduval’s specimens, morphological and genetic comparisons between modern specimens with known provenance were made to determine the origin of the *P. chloris* types. As well as *P. indetermina*, attention was also paid to the taxa that have been described as infrasubspecific entities or associated to *P. chloris* in order to ascertain their validity as well as their taxonomic and geographical boundaries. These taxa are as follows:

*Parasa minima* (Fig. 4j-l): “Identical with *chloris* H.-S., except that the green band is of about half the width … The moth is a little smaller than … *chloris*” (Dyar 1898a); “has a narrower green band than *cuernavaca*, the dark basal area is broad” (Dyar 1937).

*Parasa chloris* var. *huachuca*Dyar (1905) (Fig. 4s-u): “Differs but slightly from normal *chloris*, yet in an unexpected direction” (Dyar 1905).

*Parasa chloris* var. *cuernavaca*Dyar (1907) (Fig. 4m-o): “A form … in which the green band on the fore wings is much narrower than in specimens from the Atlantic Coast of the United States. The specimens agree with *P. minima* Schaus in markings, but are distinctly larger and more robust” (Dyar 1907).

*Parasa maysi* Schaus (1920) (Fig. 4p-r): “Near *P. minima* Schaus, which has yellow hind wings” (Schaus 1920).

### Genetic Analyses of *Parasa chloris* and Related Species

Two main lineages were recovered in the phylogenetic analyses, one comprising most of the North American specimens + *P. maysi* and the second comprising 3 further clades of mainly Central American specimens (Fig. 3). The type specimen of *P. chloris* was recovered within the clade containing specimens found from eastern USA with strong cluster support (bootstrap 86%), suggesting that this taxon is North American. The hypothesized collecting locality of the type specimen is the southern USA (Louisiana/Mississippi/eastern Texas; shaded orange area in Fig. 8), considering the original description (discussed further below).

Intraspecific pairwise distances (PWDs) of *P. chloris* ranged from 0.00% to 0.33% across the entirety of its distribution, suggesting that any minor size or morphological differences found within this range are due to variation. The interspecific PWDs between the other putative taxa ranged from 2.35% to 5.61%, with *P. maysi* appearing as the most divergent. When comparing the outgroups *P. indetermina* and *P. viridogrisea*, the former found sympatrically with *P. chloris* but morphologically distinct, the PWDs to *P. chloris* were very large, between 9.49% to 10.65% and 7.78% to 8.13%, respectively.

The second clade containing most of the Central American specimens represents 3 taxa, to which the following names can be assigned: *P. minima*, *P. Huachuca*, and *P. cuernava*ca.

Unfortunately, there was no genetic data available for topotypical specimens of *P. cuernavaca* which meant that, apart from the morphological evidence, defining the species boundary between *P. minima*, *P. maysi*, and *P. cuernavaca* remains difficult. Interestingly, the barcoded specimens from Costa Rica recovered as sister to *P. huachuca* from Arizona, USA with strong bootstrap support (88%), suggesting that this population may be more closely related to *P. cuernavaca* than *P. minima*. In addition, those from Costa Rica diverged slightly less from *P. huachuca* (2.98% to 3.33%) than *P. minima* (3.13% to 3.69%). For this reason, the barcoded specimens from Costa Rica as well as those from much of tropical Central America are here considered as *P.* cf*. cuernavaca*, albeit tentatively. Further investigations with additional specimens are needed, although this was outside the scope of the present work; it should also be noted that specimens of the *Parasa* species included in this study from Central America were generally found to be somewhat rare in the collections examined (pers. obs.).

## Taxonomy and Nomenclature of the *Parasa chloris* and Related Species

Based on the above results and detailed morphological examinations of all name-bearing types, numerous taxonomic changes are implemented herein.

### *Parasa* Moore [1860]

*Parasa* Moore [1860]: 413.

Type species: *Neaera chloris*Herrich-Schäffer [1854] by subsequent designation (Fletcher and Nye 1982: 120)

### *Parasa chloris* (Herrich-Schäffer [1854])

**Fig. 7.**
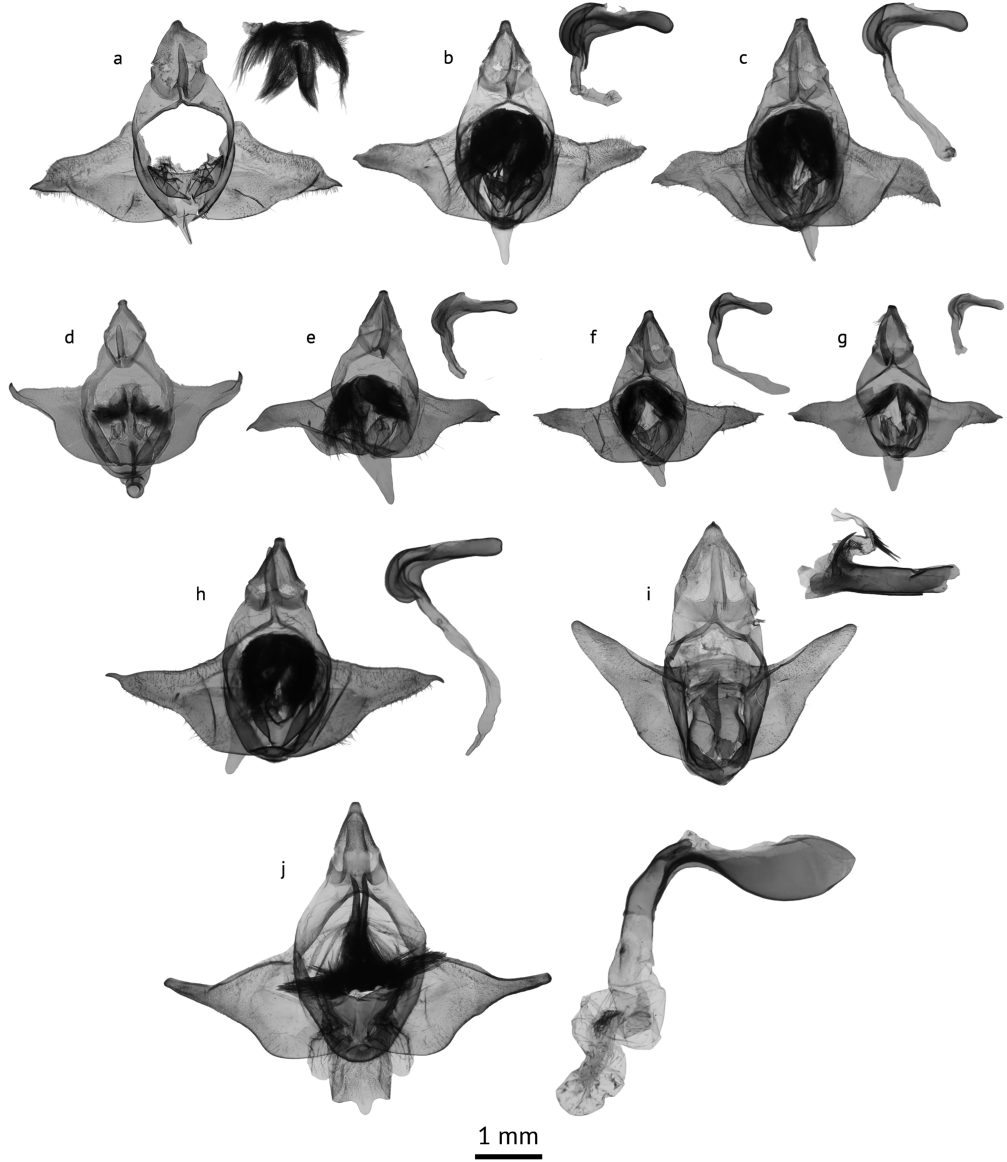
Male genitalia of *Parasa* species. Figures a-c: *Parasa chloris* (Herrich-Schäffer). a. Lectotype (note: genital capsule pest damaged and has been reconstructed for illustrative purposes), showing cluster of setae found in diaphragm. b. Virginia, Fairfax County, USA, USNMENT01918176, gen. slide no. TT255; c. Louisiana, Bossier Parish, USA, USNMENT01918180, gen. slide no. TT257; d. *Parasa cuernavaca* Dyar, Sinaloa, Mexico, USNMENT01848165, gen. slide no. USNM134534; e. *Parasa* cf. *cuernavaca*, Área de Conservación Guanacaste, Costa Rica, USNMENT01918184, gen. slide no. TT258; f. *Parasa minima* Schaus, Texas, Cameron County, USA, USNMENT01918178, gen. slide no. TT259; g. *Parasa maysi* Schaus holotype, USNMENT01918214, gen. slide no. USNM156591; h. *Parasa huachuca*, Arizona, Cochise County, USA, USNMENT01918177, gen. slide no. TT256; i. *Parasa indetermina* (Boisduval), Maryland, Patuxent Wildlife Research Center, USA, USNMENT01918182, gen. slide no. TT236; j. *Parasa viridogrisea* (Dyar), Área de Conservación Guanacaste, Costa Rica, USNMENT01918196, gen. slide no. TT245 (all USNM).

(Figs. 4a-i, 7a-c)

*Neaera chloris* Herrich-Schäffer [1854]: Tab. 37, fig. 176.

*Parasa fraterna* Grote (1881: 5).

*Neaera chloris*—Walker (1855: 1140).

*Sibine chloris*—Herrich-Schäffer (1858: 58).—Druce (1887: 211).

*Parasa chloris*—Moore (1860: 413).—Walker (1862: 171).—Grote and Robinson (1868a: 73; 1868b: X).—Stretch (1873: 209) (larval description).—Grote (1882: 17).—Smith (1891: 28).—Dyar (1891: 154; 1894: 217).—Neumoegen and Dyar (1894: 72).—Dyar (1897b: 61; 1898b: 275 (key); Dyar 1905: 186; 1906: 366).—Forbes (1923: 108).—Dyar (1937: 1114, Pl. 164, fig. h).—Fletcher and Nye (1982: 120) (fixation of genus type).

*Parasa fraterna*—Grote (1882: 17).

*Euclea chloris*—Dyar (1898a: 234; 1899: 247; 1902: 355).—Holland (1916: 365, Pl. XLVII, figs 26, 29).

*Parasa chloris chloris*—Davis (1983: 67).—Becker and Epstein (1995: 131).—Epstein and Fiesler (2023: 121).

#### Type Locality

##### Neaera chloris:

“Am. mer.”

##### Parasa fraterna:

“N.Y., Mass[achusetts]”

#### Type Material Examined

##### Neaera chloris, three syntypes

. The specimen that is the closest match to Herrich-Schäffer’s figure and with Boisduval’s hand-written label is here **designated as the lectotype** male: “nærea chloris Bd. | am. m. || EX MUSÆO | DRIS BOISDUVAL || Oberthur | Collection || Barnes | Collection || USNMENT | 01848067” (USNM). The remaining 2 specimens, a male and a female, are thus paralectotypes.

##### Paralectotype male

. “neaera chloris. | Herrich-Schaeffer | (Samml. aussereurop. | Schmetterl. No 176) || EX MUSÆO | DRIS BOISDUVAL || Oberthur | Collection || Barnes | Collection || USNMENT | 01848068”; Paralectotype female: “chloris ♀ || EX MUSÆO | DRIS BOISDUVAL || Oberthur | Collection || Barnes | Collection || Barnes | Collection || USNMENT | 01848069” (USNM).

##### Parasa fraterna, three syntypes.

1 male: “Type || ex L. | June | 17/74. || Type of: SYNTYPE | Parasa fraterna | Grote, 1881 | Det: J. Wilterding 1995 || Michigan State Univ. | Arthropod Res. Coll. | Cat#: 133898”; 1 female: Type || Type of: SYNTYPE | Parasa fraterna | Grote, 1881 | Det: J. Wilterding 1995 || Michigan State Univ. | Arthropod Res. Coll. | Cat#: 133897” (MSU/ARC); 1 female: “Parasa fraterna | Grote Type Thaxter || Parasa | fraterna | Grote | [on underside] 81.116 || SYN- | TYPE || Limac | genitalia slide | No. 354 ♀ || NHMUK 010929346” (NHMUK).

#### Description

##### Adult.

Small size, females (forewing length: 12 to 12.5 mm) larger than males (forewing length: 8.5 to 10.5 mm). Ground color of head and thorax green. Abdomen color ranging from light creamy beige to deep brown. Male antennae bipectinate in basal portion, otherwise filiform; female antennae filiform. Labial palps with short rounded third segment. Legs same ground color as abdomen. Fore wing: short, broad, triangular. Ground color brown; males often with transverse central green band of varying width which sometimes extends onto the basal area of the anal margin, distal margin of green band with strong brown outline; females always with such green band. Venation: Rs2 + Rs3 off Rs4. Fringe long. Hindwing: Ground color ranging from creamy beige to deep brown, sometimes with distal diffusion of brown scales. Fringe long. Underside of both wings paler than upper side without markings.

##### Male genitalia.

Uncus slightly shorter than tegumen, tapered, apically rounded. Tegumen broad. Gnathos straight, slender, apically pointed or rounded. Juxta flat, rounded at apex; transtilla with paired cluster of setae. Vinculum short and broad, rounded. Valva broad at base, triangular in shape or with medial constriction on proximal margin; distally narrowing and often with sharp point. Phallus short, right-angled with medial lateral lobes; membranous in basal portion. Vesica long, membranous without cornuti.

##### Larva.

Nettle-type larvae. Coffin-shaped with dorsal hump towards anterior end, final section of posterior end with pointed tail. Anterior end with 3 raised humps with pair of pale stinging spine clusters, the largest arising at the top of the hump; posterior end with 2 prominent pairs of spine clusters; smaller pairs of spine clusters found along the dorsolateral lines, and above the spiracles laterally. Ground color brown to orange, with numerous indistinct wavy lines along the body.

##### Diagnosis.

Forewing length male: 8.5 to 10.5 mm (*n* = 8); female: 12 to 12.5 mm (*n* = 5). *Parasa chloris* and *P. huachuca* possess the broadest green forewing bands compared to all other species in this group. The latter is a paler brown, larger moth with reduced brown distal margins on the hindwing and the 2 species are not found in sympatry. In the male genitalia, the valves of both species are ca. one-third longer than the other species in the group. Compared to *P. huachuca*, the apex of the valve is broader in *P. chloris*, whereas it is strongly tapered, forming a hooked denticuate tip in the former.

##### COI divergence.

The COI divergence within *P. chloris* was 0.00% to 0.33% (*n* = 10) across its range. Its nearest neighbour was *P. maysi*, with intraspecific PWDs of 2.35% to 2.80%.

##### Distribution.

This species has a broad range across temperate forests of north and eastern USA, likely limited westwards by the drier, prairie plains; westernmost points in Arkansas and eastern Texas.

##### Notes.

*Parasa fraterna* was described by Grote based on an unknown number of specimens collected by Roland Thaxter with other examples “in Mr. Tepper’s collection.” The collection of Frederick Tepper, an avid American entomologist, contained 12,000 Lepidoptera and was donated to the MSU/ARC in 1889 (Wilterding 1997). Two syntypes (1 male and 1 female) of *P. fraterna* were listed in a type catalogue of this collection by Wilterding (1997) and are here illustrated (Fig. 4d-e). A single female syntype (ex. Thaxter) is also present in NHMUK (Fig. 4f), the institution having purchased the first half of Grote’s private collection, consisting of 3,247 specimens and including numerous type specimens, in 1881 (accession number B.M. 1881-116).

### *Parasa cuernavaca* Dyar (1907) stat. rev.

(Figs. 4m-o, 7d-e)

*Parasa chloris* var. *cuernavaca*—Dyar (1907: 565).

*Parasa cuernavaca*—Dyar (1937: 1114).—Becker and Epstein (1995: 131).

*Parasa minima*—Epstein and Corrales (2004: 7).

#### Type Locality

“Cuernavaca, Mexico”

#### Type Material Examined

##### Lectotype.

Male: “Cuernavaca | Mex || Collection | Wm Schaus || July | ’06 || Type | No. 10337 | U.S.N.M. || LECTOTYPE ♂ | MS | By M.E. Epstein || USNMENT | 01918212” (USNM).

##### Diagnosis.

Forewing length male: 10 to 11 mm (*n* = 4). Of the species with narrow green forewing bands and yellow hindwings, *P. cuernavaca* is most similar to *P. minima*, although the latter is a smaller insect with paler more uniform hindwing coloration while in the male genitalia, the apex of the valve in the latter is smaller and finer.

##### COI divergence.

No specimens of *P. cuernavaca* from its type locality of Western Mexico could be sequenced. However, 5 specimens from Costa Rica were sequenced (herein considered as *P.* cf*. cuernavaca* until further work is completed) and revealed interspecific PWDs of 0.00% to 0.16% (*n* = 5) and intraspecific PWDs of 2.98% to 3.33% to its nearest neighbour *P. huachuca*.

##### Distribution.

This species is distributed from the coastal plains of western Mexico to the semi-arid highlands and temperate forests of the Sierra Madre del Sur. It is possible that this species and *P. minima* are separated by the Sierra Madre Oriental Mountain range, although further work is needed to understand their geographical boundaries. The Central American specimens distributed from east of the Isthmus of Tehuantepec in Mexico, southwards through the western edge of the Sierra Madre de Chiapas highlands and the Talamancan montane forests in Costa Rica, are here referred to as *P.* cf*. cuernavaca*. There are some differences in the genitalia when compared to typical *P. cuernavaca*, notably the longer gnathos, and the finer apex of the valve. Their identity could not be accurately verified due to a lack of museum specimen data, however, based on genetic results, adult morphology of the dissected and barcoded specimens and highland distribution, it is likely these specimens are more closely related to *P. cuernavaca* than *P. minima*. The presence of a photographed record on iNaturalist from near Turbaco, northern Columbia suggests that the species crosses into the northerly point of South America. Additional investigations are required to test the specific boundaries of all Central American taxa.

### *Parasa huachuca* Dyar (1905) stat. nov.

(Figs. 4s-u, 7h)

*Parasa chloris* var. *huachuca—*Dyar (1905: 186);

*Parasa chloris* var. *huachuca*—Dyar (1907: 565).

*Parasa chloris huachuca*—Davis (1983: 67).—Becker and Epstein (1995: 131).—Epstein and Fiesler (2023: 121).

#### Type Locality

“Palmerlee, Cochise Co., Arizona”

#### Type Material Examined

Holotype. Male: “Parasa | huachuca | Type Dyar || Palmerly | Cochise Co. | VII. Ariz. || Collection | BrklynMus || USNMENT | 01978951” (USNM).

##### Diagnosis.

Forewing length male: 10.5 to 11.5 mm (*n* = 9); female: 13 to 15 mm (*n* = 5). *Parasa huachuca* is most similar to *P. chloris* but the latter is in general a smaller moth with a greater degree of brown diffusion along the distal portion of the hindwing. Differences in the male genitalia are discussed under *P. chloris*.

##### COI divergence.

Interspecific PWDs of *Parasa huachuca* were 0.00% to 0.16% (*n* = 4), and intraspecific PWDs to its nearest neighbour, *P.* cf*. cuernavaca*, were 2.98% to 3.33%.

##### Distribution.

This species appears to be confined to the forested Sky Island mountains in southern Arizona and the extreme north of Mexico.

##### Notes.

*Parasa huachuca* is here raised to species level. The taxon was closest in genetic distance to *P.* cf. *cuernavaca* (2.98% to 3.33%) and was considerably different in DNA to *P. chloris*, at a COI divergence of 3.57% to 4.44%. Additional morphological distinctions between *P. huachuca* and *P. chloris* can be found under the *P. chloris* diagnosis.

### *Parasa maysi* (Schaus, 1920)

(Figs. 4p-r, 7g)

*Parasa maysi* Schaus (1920: 146).

*Parasa maysi*—Dyar (1937: 1114).—Becker and Epstein (1995: 131).

#### Type Locality

“Cayuga, Guatemala”

#### Type Material Examined

##### Holotype.

Male: “Parasa | maysi | type Schs || Cayuga| Guat || Sept. || Schaus and | Barnes | coll || Type No. | 22484 | U.S.N.M. || USNMENT | 01918214” (USNM).

##### Diagnosis.

Forewing length male: 9 mm (*n* = 3); female: 11 to 11.5 mm (*n* = 3). *Parasa maysi* can be easily distinguished from all other species due to their dark brown hindwings, and the extremely narrow green band of the forewing. The genital capsule is most similar to *P. minima* but in the latter, the diaphragm is larger and more setose.

##### COI divergence.

Interspecific PWDs of *P. maysi* was 0.46% (*n* = 2). Based on the COI region, its nearest neighbour was *P. chloris*, with intraspecific PWDs of 2.35% to 2.80%.

##### Distribution.

**Fig. 8.**
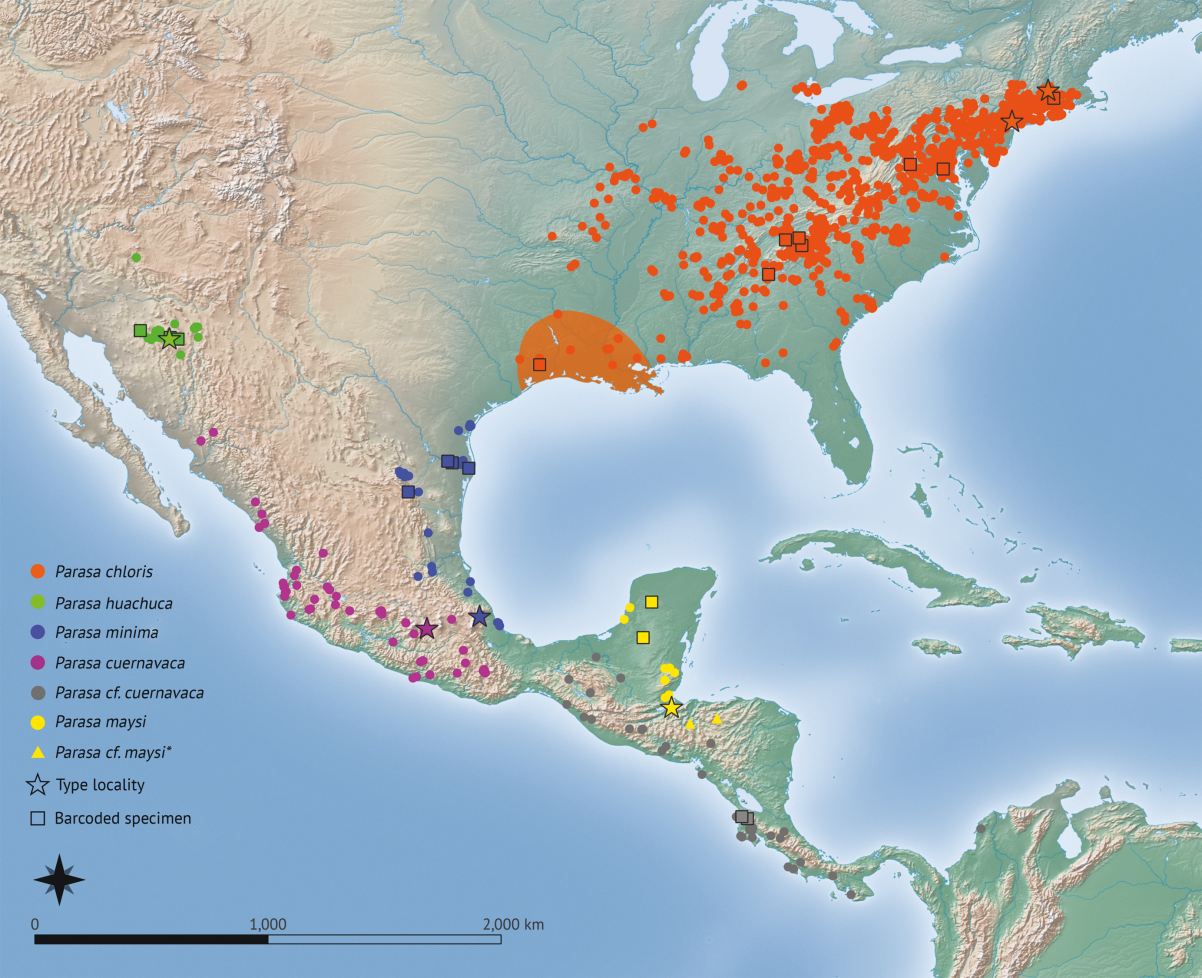
Distribution of *Parasa chloris* and related species, including the likely type locality of *P. chloris* (shaded orange area) based on genetic and morphological evidence. Circles and triangles represent specimen data from iNaturalist and natural history museum collections, outlined stars represent type localities of each species (note the stars for *P. chloris* represent the type localities of *Parasa fraterna*, a junior synonym of *P. chloris*), and outlined squares are specimens which are represented in the maximum likelihood phylogeny in Fig. 3. The shaded orange area represents the proposed collecting locality of the *P. chloris* type specimens. *Due to uncertainty in species geographical boundaries in Central America, these points are tentatively identified as *P. maysi*. iNaturalist distribution records were downloaded as of November 2024, with filters selecting for “Research Grade” and imaged records, so they could be cross-referenced. Records were downloaded from pages listed as *Parasa chloris* and *Parasa minima* at the time. Point data can be found in Supplementary Information S2.

Known from northern Guatemala, the Yucatan Peninsula in Mexico, Belize and northern Honduras. Two specimens on the distribution map (Fig. 8) were marked as *P.* cf*. maysi*, as they were iNaturalist records and the hindwing color could not be seen to confirm identification.

### *Parasa minima* Schaus (1892)

(Figs. 4j-l, 7f)

*Parasa minima* Schaus (1892: 322).

*Parasa minima*—Dyar (1906: 366; 1937: 1114).—Becker and Epstein (1995: 131).—Epstein and Corrales (2004: 7).—Epstein and Fiesler (2023: 121) (partim).

*Euclea minima*—Dyar (1898a: 234).

*Parasa chloris* var. *minima*—Dyar (1907: 565).

#### Type Locality

“Coatepec, Mexico”

#### Type Material Examined

Holotype. Female: “Parasa | minima | Type. Sch. || Coatepec, | Mex. || Collection | W. Schaus || Type | No. 9108 | U.S.N.M. || USNMENT | 01848166” (USNM).

##### Diagnosis.

Forewing length male: 8 to 8.5 mm (*n* = 3); female: 10 mm (*n* = 2). Adult *P. minima* specimens are considerably smaller when compared with other species. It is most comparable in size to *P. maysi*, although *P. minima* can be readily distinguished due to its very pale hindwing coloration compared to the very dark hindwings of *P. maysi*. Differences in the male genitalia are discussed under *P. maysi*.

##### COI divergence.

The COI divergence within *P. c. minima* ranged from 0.15% to 1.87% (*n* = 6). Its nearest neighbour was *P. chloris*, with a genetic distance of 2.35% to 3.95%.

##### Distribution.

This species is found from the lower Rio Grande Valley in south-eastern Texas extending along the coastal tropical forests of eastern Mexico to the forested regions of the Isthmus of Tehuantepec.

### *Parasa indetermina* (Griffith and Pidgeon, 1832*nec* Boisduval)

(Figs. 5, 7i)

*Limacodes indeterminus* Griffith and Pidgeon (1832: 789, Pl. 103, fig. 8).

*Limacodes viridus* Reakirt (1864: 251). **syn. rev.**

*Callochlora vernata* (Packard 1864: 339).

*Phalaena cippus* sensu Smith & Abbott (*nec* Cramer)—(1797: 145), Tab. LXXIII (larvae only).—Duncan (1841: 177, Pl. XXI, fig. 1) (larva only).—Harris (1842: 303).

*Parasa chloris* (sensu auct.)—Grote and Robinson (1868a: 73; 1868b: X).—Stretch (1873: 209, Pl. 8, fig. 17).—Gerhard (1878: 57).—Mann (1879: 271).—Grote (1881: 5).—Grote (1882: 17).—Edwards and Elliot (1883: 128).—French (1885: 161).

*Callochlora chloris*—Wetherby (1875: 368).

*Parasa viridus*—Smith (1891: 28).—Dyar (1891: 154).

*Euclea indetermina* (Boisduval)—Dyar and Doll (1893: 311).—Dyar (1894: 214).—Neumoegen and Dyar (1894: 68).—Dyar (1897a: 10; 1899: 247; 1902: 355).—Holland (1916: 365, Pl. XLVII, figs 10, 15).

*Parasa indetermina* (Boisduval)—Dyar (1898b: 275 (key); 1906: 366).—Forbes (1923: 107).—Dyar (1937: 1114).—Davis (1983: 67).—Epstein and Fiesler (2023: 121).

#### Type Locality

Limacodes viridus: “Philadelphia”

Callochlora vernata: “New York” “Phil.[adelphia]”

#### Type Material Examined

##### Limacodes viridus, two syntypes.

1 male: “Parasa Viridus | Phila Pa. Reak. | orig. Types coll. Reakirt || Lim. Viridus | Reak. | Phila. Pa. | orig. coll. | Type Reakirt || SYNTYPE ♂ | Parasa viridus Reakirt, 1864 || Type status verified | in orig. desc. JKW ’24 || ‘Parasa viridis Reak. | Pa., Phila.’ | ‘One of these is the type’ | Strecker Colln. 29404 | Field Museum Nat. Hist. || PHOTOGRAPHED | Jessica Wadleigh 2024 | CIL Request || FMNHINS | 4518590 | FIELD MUSEUM | Pinned”; 1 female: “Parasa Viridus | Phila Pa. Reak. | orig. Types coll. Reakirt || L. Viridus | Reak. | Phila. Pa. | orig. coll | Type Reakirt || Lepidoptera Type | Photograph No. 205 | Field Museum || FMNH-INS | 0000-095-028 || PHOTOGRAPHED | Allie Stone 2012 | KE Emu catalog” (FMNH).

##### Callochlora vernata, two possible syntypes.

1 male: “C. vernata | n.y. Pack. | United States. | Packard Coll. || MCZ | Syntype | 36358 || MCZ-ENT | 00036358” (MCZ); 1 male: “81.116” (NHMUK).

##### Diagnosis.

Forewing length male: 11 to 12.5 mm (*n* = 8); female: 14 to 15 mm (*n* = 5). When compared to *P. chloris*, *P. indetermina* is slightly larger in size with noticeably rounder, broader forewings. A large dark brown semi-circular marking is always present (even in worn specimens) along the forewing margin between veins Rs4 and M3. In the male genitalia, the cluster of setae diagnostic of *P. chloris* is absent, and the phallus is straight with cornuti in the vesica.

##### COI divergence.

*Parasa indetermina* diverged from *P. chloris* by 9.49% to 10.65%.

##### Distribution.

This species is distributed throughout much of the temperate forests of eastern USA from Illinois to Long Island in the north to Louisiana, Texas and Florida in the south (Wagner 2005).

##### Notes.

The name *indetermina* has been credited to Boisduval in the American literature since Dyar and Doll (1893) but the authorship is incorrect and it is not entirely apparent why; the correct authorship here is considered Griffith and Pidgeon (1832). An unidentified limacodid larva was illustrated as Figure 8 on Plate 87 of Guérin-Méneville’s ([1832]) *Iconographie du Règne animal de G. Cuvier* and labeled “*Chen*[*ille*] *de Lim*[*acodes*] *indéterminée*” (see Fig. 6 herein) while in the text section accompanying the plates, it was stated that the larva was found in “l’Amerique du Nord.” As McDunnough (1918) remarked, this is “certainly no specific name,” further supported by the name “*Limacodes* sp. *ignota* [= unknown in latin]” appearing in the index of Guérin-Méneville’s work and the only one (in 16 pages of names) without an author.

Due to the renown and distinction of Cuvier’s ([1816]) *Le Règne animal*, a translation of this work incorporating changes and additions by various experts was undertaken by Griffith (1824 to 1835) at around the same time as Guérin-Méneville’s *Iconographie* (Cowan 1969). The insect section of Griffith’s *Animal Kingdom* (Volume 15, published in 1832) was written by Griffith and Pidgeon and in it, a perfect copy of Guérin-Méneville’s Plate 87 was made by Westwood and included as Plate 103. Early authors dealing with Guérin-Méneville’s and Griffith and Pidgeon’s publications (e.g., Harris 1842) may have regarded the latter as a mere English translation of the former, and further assumptions may have been made that Griffith and Pidgeon’s publication took priority based on the imprint date on the title pages. Westwood’s copy of the plate in Griffith and Pidgeon never preceded Guérin-Méneville’s originals (Cowan 1971), however, the name was latinized by Griffith and Pidgeon (1832) to *Limacodes indeterminus* and it has been suggested (McDunnough 1918) that authorship of this name should be credited to Griffith and Pidgeon. No description of the larva was provided in either publication, although in Griffith and Pidgeon, it was listed in the index as being the larva of *Limacodes delphinii*Guérin-Méneville [1832] (note once more the authorship of this taxon is not Boisduval sensu auct.), which Harris (1842), incorrectly citing Guérin-Méneville rather than Griffith and Pidgeon, added could not be the case as the larva does not feed on *Delphinium* (Ranunculales: Ranunculaceae).

From a nomenclatural perspective, there may be reason to consider *Limacodes indeterminus*Griffith and Pidgeon (1832) a *nomen nudum* (or even a *nomen dubium*) but by stating that it was the larva of *L. delphinii*, the name conforms to Article 12 of the ICZN and is herein considered valid. It was not until Dyar and Doll (1893) that the highly distinctive larva figured by Guérin-Méneville (as Boisduval) was first associated with the *Parasa* complex and that it was in fact the *chloris* sensu auct. (e.g., Grote 1881).

*Parasa vernata* was described by Packard based on material he examined from New York in Grote’s collection and from Philadelphia in “Coll. Ent. Soc.” the Collection of the Entomological Society. After checking the collections of the NHMUK, a male specimen from Grote’s private collection (B.M. 1881: 116—see above under *P. fraterna*) was found (Fig. 5b). No locality data were provided on the label, and so it is unknown whether this was collected in New York, although Grote’s own type labels also lacked locality data (Fig. 4d-f). For this reason, this specimen is here considered a possible syntype of *P. vernata*. Locating the other type specimen(s), listed from the Collection of the Entomological Society (now ANSP), was less clear. The ANSP exchanged the majority of their Lepidoptera collection with CMNH in the 1960s, although this was poorly documented (Jason Weintraub, pers. comm.) and no specimens marked as types or with a Packard provenance were found in CMNH. The specimen listed as a type by Henshaw (1887) in MCZ is illustrated in Fig. 5a. Parts of Packard’s Lepidoptera collection ended up in the PEM where it was partly damaged or destroyed (Horn et al. 1990) before being transferred to MCZ in 1885 (Jason Weintraub, pers. comm.). Although possible that Packard retained an ANSP specimen he studied as part of his description, the label data of this specimen is inconsistent with the original description in both type locality and collection (Fig. 5a) and thus the type status of this specimen remains doubtful.

In the description of *Parasa viridus*, Reakirt described both a male and female from Philadelphia based on specimens presumably in his own collection. Reakirt’s collection was incorporated into that of Herman Strecker, which was then purchased by the FMNH in 1908 (Brown 1964). Two syntypes, one of each sex, are illustrated here (Fig. 5c-d).

Finally, based on the male genitalia, it is here posited that *P. indetermina* may not be as closely related to *P. chloris* as typically thought. *Parasa chloris*, as well as closely related species such as *Parasa viridogrisea* (Fig. 7j) and the other species discussed in this article possess distinctive characteristics of the male genitalia. Firstly, and most diagnostic, the central transtilla possesses a large pair of clusters of setae (see Fig. 7a for a flattened view of this structure). Such character has not been observed in any other *Parasa* from Asia or the Afrotropics (pers. obs.), further questioning the global relatedness of the supposedly pantropical genus. Secondly, the phallus is right-angle shaped, curving strongly in the distal portion with medial lateral lobes. This cluster of setae is completely absent in *P. indetermina* (Fig. 7i), and the phallus of this species is straight, with a long curved posterior process and cornuti in the vesica; the large differences between these sympatric species further solidify their separation.

## Discussion

This research demonstrates the first, detailed review of the nomenclature and identification of *Neaera chloris*, the type species of *Parasa*, thus accurately defining the genus as a whole and aiding future revisional work. Based on the above morphological and genetic evidence, the type specimen of *P. chloris* is formally recognized for the first time as originating from eastern USA, more specifically the southern states, and not South America as suggested in the original description. Through examination of all relevant name-bearing types, the convoluted taxonomic history of *P. chloris* and its relationship with *P. indetermina* has been resolved, with *Limacodes viridus* now considered a junior synonym of the latter rather than the former. Several other nomenclatural changes have been implemented within this group, clarifying their long-standing taxonomic confusion. Finally, putative phylogenetically informative characters of the male genitalia have been identified and are discussed briefly, which require further investigation in other members of *Parasa*. It should also be noted that the female genitalia were not investigated here, which may reveal further important diagnostic characters.

Citizen science data on the online platform iNaturalist has proved incredibly useful for the most part in understanding the distribution of *Parasa* species but due to the resting position of these moths with the diagnostic underside being hidden, it is sadly not a replacement for physical examination of specimens. Additional specimens of *P. maysi*, *P. minima*, and especially *P. cuernavaca* from Central America need to be studied to determine the spatial limits of these 3 species, and whether a further species near *P. cuernavaca* (and currently without a name) exists in southern Central America.

This study emphasizes once more the need for efforts in tracing and identifying type material. It is surprisingly not uncommon for taxonomic revisions to be undertaken without examination of type specimens, which can lead to erroneous decisions about nomenclature in accordance with ICZN conventions (Hausmann et al. 2016). This is despite the fact that in many cases, and especially in modern species descriptions, type material can be relatively easy to locate through physical and online literature searches. There is also an increase in natural history museum collections being digitized, whereby type specimen images are available online and accessible to all (Wilson et al. 2023). On the other hand, it is sometimes known when types are definitively lost or destroyed (e.g., ZMH in 1943 during the Second World War or NMUFRJ in 2018 as a result of fire), and hence neotype designations can confidently be made. Despite this, there are likely also countless examples of unidentified type specimens present in museum drawers, unbeknownst to curators due to poor historical documentation and/or having never originally been labeled as such (as was the case in *P. chloris*). These problems are amplified further in groups that have not received revisionary work since their description, which can sometimes be a hundred years or more.

In the case of Herrich-Schäffer’s types, which are generally poorly documented and described from many different collections, it is somewhat understandable that authors consider type material as lost or unknown (e.g., Fiebig et al. 2023, Häuser et al. 2003). Despite this, basic literature searches led to the discovery of the type material of *P. chloris*, emphasizing the need for taxonomists to make their best efforts to locate types before defining them as permanently lost (and worse, designating an invalid neotype). Such actions are even more important when dealing with the type species of the genus, whose taxonomic status holds additional weight to systematic hypotheses.

Finally, this work supports calls for integrating name-bearing specimens into molecular revisions of species hypotheses where possible (e.g., Puillandre et al. 2011, Strutzenberger et al. 2012, Sánchez et al. 2022). This allows genetic clusters to be more confidentially linked to available names, establishing the identity of described species and elucidating taxonomic issues such as synonyms (Puillandre et al. 2011), determining type localities (Pfeiler 2018), and identification of cryptic taxa (Hausmann et al. 2016). While sequencing DNA of old Lepidoptera type material was previously costly and time-consuming (Hausmann et al. 2009, Lees et al. 2010), advances in NGS protocols employing multiplex PCR primers have allowed for successful amplification of the COI gene (Speidel et al. 2015, Prosser et al. 2016, D’Ercole et al. 2021). This has made it feasible to obtain sequence information required to establish the identity of type specimens in an objective manner. In the case of *P. chloris*, it is shown here that even short COI sequences can be taxonomically informative for solving such issues, supporting similar conclusions to many others (Hajibabaei et al. 2006, Meusnier et al. 2008, Thomsen et al. 2009, Shokralla et al. 2011, Mutanen et al. 2015). The high value of extracting genetic data from historic types is thus emphasized in this study.

## Supplementary Material

Supplementary material is available at *Annals of the Entomological Society of America* online.

saaf016_suppl_Supplementary_Materials

## Data Availability

Data associated with this research can be found at the following URL: https://figshare.com/projects/Defining_a_genus_An_investigation_into_the_type_species_of_Parasa_Moore_1860_Lepidoptera_Limacodidae_/237038 The GenBank accession number for the single newly sequenced type specimen is PV365262.

## Notes

Version of Record, first published online 14 July 2025, with fixed content and layout in compliance with Art. 8.1.3.2 ICZN.
