# Supplementary material for: On the identity of the type species of Parasa (Lepidoptera: Limacodidae): investigations into the Nearctic Parasa chloris and related taxa
Source: Ann Entomol Soc Am. 2025 Jul 14;118(4):276–89. doi: 10.1093/aesa/saaf016 (PMC12285581; doi:10.1093/aesa/saaf016)
Supplement: saaf016_suppl_Supplementary_Materials [file saaf016_suppl_supplementary_materials.zip › saaf016_suppl_Supplementary_Materials_S3_.docx]

**S3: Efforts made to trace the type material of *Parasa chloris***

Herrich-Schäffer amassed a large collection which is now scattered across various European museums (Horn *et al*. 1990), and since the majority of species he described were based on specimens from collections aside from his own, in some cases the type material remains unaccounted for (Häuser *et al*. 2003). Within the text section of *auss. Schmett.* (Herrich-Schäffer [1858]), the depository of the type specimen is not stated (Fig. 2). This information is only provided in the plate wrappers, of which just two complete copies are known; one each in the MfN and NHMUK (Häuser *et al*. 2003). Upon examining the NHMUK copy (Fig. 1), it was ascertained that the description of *P. chloris* was based on material belonging to the collection of Jean-Baptiste A. D. Boisduval (denoted with “Coll. Boisd.”).

Boisduval (1799–1879) was a renowned French lepidopterist whose important collection was acquired in 1876 by Charles Oberthür (1819–1895), another French entomologist who at one point owned the second largest private collection of butterflies in existence (McDunnough 1916). Upon his death, the collection was split up, and although a large part of it ended up in NHMUK (Horn *et al*. 1990), the remainder is now scattered throughout museum collections making the ability to track down certain specimens more difficult. However, Oberthür labelled all of Boisduval’s specimens with a printed label that reads “EX-MUSEAO | Dris. Boisduval” (Talbot 1943), making them identifiable. Upon examination of the American *Parasa* specimens in the main collection of NHMUK, potential type material could not be identified.

Further research revealed that the American lepidopterist William Barnes (1860–1930) acquired many North American butterfly specimens from Oberthür’s collection in a sale after his death in 1924 (Calhoun, 2006; 2019) and added a yellow label with “Oberthur [sic] | Collection” printed in red ink to all of the specimens from this collection. Barnes’ collection of nearly half a million specimens was purchased by the U.S. government in 1930 and currently resides in the USNM, all specimens from this collection clearly diagnosed with a printed label “Barnes | Collection” in red ink. Given this information and assuming that the type material of *P. chloris* followed this route from Boisduval to Oberthür to Barnes, it should a) bear the corresponding labels from each of these collections, and b) now be held in the USNM. Accordingly, three specimens of *P. chloris* matching this provenance trail were identified (see Figs. 4a−c) and are herein considered syntypic.

When comparing these three specimens to Herrich-Schäffer’s original illustration (shown in Fig. 1), the male specimen USNMENT01848067 (Fig. 4a) is the closest match (for example, in the brightness of both the distal edge of the post-medial band and the distal margin of the forewing, the shape of the antennae and the posterior end of the abdomen). In addition, the handwritten label is in Boisduval’s hand (see Correa-Carmona et al. 2021: fig. 2 for comparison). It should be noted that in the spelling of the genus *Nærea*, the ligature is an ‘a’ and ‘e’ and not the other way around as published by Herrich-Schäffer ([1854]). Specimen USNMENT01848068 (Fig. 4b), another male, possesses a label that is in a different hand, most likely Oberthür’s, and refers to the figure of *P. chloris* in Herrich-Schäffer ([1854]) (“no 176”) suggesting that it must have been added later. Specimen USNMENT01848069 (Fig. 4c), a female, carries a label possibly in Boisduval’s hand. With regard to the type status of these three specimens, the recommendation of Häuser *et al*. (2003) is followed here and they are all considered as syntypes. This is because Herrich-Schäffer gave no indication about the number of specimens examined for any of the taxa he described throughout *auss. Schmett.*. In order to fix the identity of this taxon, and in accordance with ICZN Article 74.7 Recommendation 74B, specimen USNMENT01848067 is here designated the lectotype of *Neaera chloris*.

**References**

Calhoun JV. 2006. More on *Melitaea isomeric* Boisduval Le Conte: the discovery of Boisduval specimens of *Chlosyne nycteis* (Doubleday). News Lepid. Soc. 48(2):56–59.

Calhoun JV. 2019. From oak woods and swamps: The butterflies recorded in Georgia by John Abbot (1751–C.1840) based on his drawings and specimens. J. Lepid. Soc. 73(4):211–256. <https://doi.org/10.18473/lepi.73i4.a8>

Correa-Carmona Y, Giusti A, Haxaire J, et al. 2021. Three new species of the *Xylophanes crotonis* species-group (Lepidoptera: Sphingidae) from Colombia and a neotype designation for *Xylophanes aristor*. Eur. J. Entomol. 118:64–81. <https://doi.org/10.14411/eje.2021.009>

Häuser CL, Bartsch D, Holstein J, et al. 2003. The Lepidoptera type material of G. A. W. Herrich-Schäffer in the Staatliches Museum für Naturkunde, Stuttgart. Stuttgarter Beiträge Zur Naturkunde Serie A (Biologie) 657:1–78.

Herrich-Schäffer GAW. 1850–1858. Sammlung neuer oder wenig bekannter aussereuropäischer Schmetterlinge. G.J. Manz. <https://doi.org/10.5962/bhl.title.49772>

Horn WW, Kahle I, Friese G, et al. 1990. Collectiones Entomologicae: eine Kompendium über den Verbleib entomologischer Sammlungen der Welt bis 1960. Akademie der Landwirtschaftswissenschaften der DDR.

McDunnough J. 1916. Chasing Butterflies for Money. Pop. Sci. Monthly 6(88): 872−875.

Talbot G .1943. XI. On some Types of Pieridae described by Boisduval. J. Nat. His. 10(62):136–139.
