# Supplementary material for: On the identity of the type species of Parasa (Lepidoptera: Limacodidae): investigations into the Nearctic Parasa chloris and related taxa
Source: Ann Entomol Soc Am. 2025 Jul 14;118(4):276–89. doi: 10.1093/aesa/saaf016 (PMC12285581; doi:10.1093/aesa/saaf016)
Supplement: saaf016_suppl_Supplementary_Materials [file saaf016_suppl_supplementary_materials.zip › saaf016_suppl_Supplementary_Materials_S4.docx]

**S4: Detailed account of how *Parasa chloris* has been treated throughout the literature, including comments on Boisduval’s potential mislabelling**

To establish a more precise type locality, it is important to determine how *P. chloris* has been treated throughout the literature. A few years after Herrich-Schäffer’s description of *P. chloris*, Morris’ (1860) “*Catalogue of the described Lepidoptera of North America*” did not include this species. He did however include two other Herrich-Schäffer species which were originally described from “*Am. bor.*” (“America borealis”), suggesting that he had seen Herrich-Schäffer’s [1858] publication and took the type locality of *P. chloris* as South America.

The monotypic genus *Callochlora* Packard, 1864 (Lepidoptera: Limacodidae) was described with the type species *C. vernata* Packard, 1864 (Figs. 5a−b) from New York and Philadelphia, a moth that is highly reminiscent of *P. chloris*. Grote (1865) synonymised *C. vernata* with *Limacodes viridus* Reakirt, 1864 (Figs. 5c−d) from Pennsylvania, which was described one month prior, and transferred *L. viridus* to *Callochlora*. In Grote & Robinson’s (1868a) publication on North American Lepidoptera, they stated that *chloris* “Belongs to *Parasa*” and synonymised *C.* *viridus* with *P. chloris*, suggesting therefore that if the type locality was not North America, then at the very least its distribution extended from “South America” into North America.

This conclusion was followed by Grote & Robinson (1868b: a North American publication that included “the Continent lying North of Mexico”), Stretch (1873: who stated *P. chloris* as being from Pennsylvania and New York based on its junior synonyms at the time), Gerhard (1878), Mann (1879: work on North American species), Grote (1882: covered the “North American Continent, north of Mexico”), French (1885), Smith (1891: “faunal region north of the Mexican boundaries”), and Dyar (1891: described habitat as “Middle States”) amongst others. Within this period, the species *Parasa fraterna* Grote, 1881 (Figs. 4d–f) was described, with larvae as being “quite distinct” from that of *P. chloris* based on information provided by Grote’s colleague, Frederick Tepper.

Interestingly, *Parasa chloris* was treated in the genus *Sibine* by Druce (1887) within the Lepidoptera section of Godman & Salvin’s *Biologia Centrali-Americana*, citing the species as being from “South America” based on the original description of Herrich-Schäffer. Druce (1887) also commented that “This genus [*Parasa*] is very widely distributed throughout Asia, Africa, and Madagascar; it also inhabits the New World”, beginning the general consensus that *Parasa* is pantropical. Kirby (1892) elaborated further by listing 54 species under *Parasa* from throughout the global tropics whilst including *P. chloris* as being from the “United States”; from here onwards, authors no longer cited the species as being from South America as per its original description.

Further confusion surrounding the treatment of *P. chloris* has been due to its association with another phenotypically similar species. *Parasa indetermina* (Figs. 5e–f) was originally described in the genus *Limacodes* Berthold, 1827 (Lepidoptera: Limacodidae) from an illustration of its distinctive larva which is very different to *P. chloris* larvae despite the similarity of the adults (Fig. 6) (see Wagner (2005) for detailed larvae descriptions). Moreover, these two species occur in sympatry in eastern USA, and their three junior synonyms (*C. vernata*, *L. viridus* and *P. fraterna*) have been repeatedly swapped between the two species during the 19^th^ Century.

Mislabelled specimens are a well-known artifact in analyses of historic museum collections and whether it is due to poor record-keeping by the collector on their long and arduous voyage, or the ever-changing geopolitical boundaries in the 19^th^ Century (Lichy (1968) suggested that Boidsuval adopted the name “Guatemala” for all of Central America from the southern border of Mexico to Colombia), it is of no great surprise.

Given *Parasa chloris*’ distribution and association throughout the North American continent, there is an indication that the locality data on the lectotype specimen was incorrect. Much like all collectors of his day, Boisduval’s entomology collection would likely have been amassed from various sources, such as friends and acquaintances who went on voyages, or purchases and exchanges with other collectors. For instance, in the preface to *Histoire Naturelle des Insectes Species Général des Lépidoptéres*, Boisduval (1836) stated that specimens from Guyana came from Lacordaire, those from Chili and Bolivia from d’Orbigny, and Mexico from Madame Salle. In terms of the *P. chloris* types, the lack of further information makes it impossible to pinpoint exactly who and where the specimens came from before entering Boisduval’s collection.

The use of “Amérique méridionale” implies that these specimens were from South America. In Boisduval’s own publications, he clearly distinguished between North and South America with various terms. For instance, Boisduval & Le Conte ([1833]) referred to North America as “Amérique du Nord” or “Amérique septentrionale”, as well as specifically to the United States of America (“Les États-Unis”) whilst southern USA was referred to as “les parties méridionales des États-Unis”. In Boisduval (1836), clear distinctions between “Amérique méridionale” and “Amérique septentrionale” were made throughout, with many of the species now known to be restricted to either continent being correctly associated to their respective biogeographic realms. Other French naturalists of the early 19^th^ century also used such terms to denote these regions (e.g., d’Orbigny 1835–1847).

However, there are numerous cases of incongruence between the “South American” type localities and their current-day known distribution in previous literature (Table S4.1). For instance, *Castnia briareus* Houlbert, 1917 and *C. hecate* Herrich-Schäffer, [1854] (Lepidoptera: Castniidae) were both described from “l’Amerique méridionale” but are known to be endemic to Mexico and Hispaniola respectively. In botany, the fern *Asplenium unilobum* Poiret, 1812 (Polypodiales: Athyriaceae) was originally from “Amérique méridionale” but the type specimen was subsequently confirmed by Morton (1973) as originating from Santa Domingo, Dominican Republic.

Table S4.1. Localities of Lepidopteran species given by Boisduval and others as “Amérique méridionale” [=South America] that are inconsistent with current-day distribution knowledge of the species.

| **Original combination** | **Current combination** | **Distribution provided by Boisduval or others** | **Current known distribution** |
| --- | --- | --- | --- |
| *Neaera chloris* Herrich-Schäffer, [1854] | *Parasa chloris* | “Amérique méridionale” (Herrich-Schäffer [1854], based on Boisduval label) | North and Central America |
| *Castnia hecate* Herrich-Schäffer, [1854] | *Ircila hecate* | “Amérique méridionale” (Herrich-Schäffer [1854] based on Boisduval label) | Endemic to Hispaniola (Vinciguerra 2008) |
| *Castnia briareus* Houlbert, 1917 | Subspecies of *Athis inca* (Walker, 1854), thus *Athis inca briareus* (Houlbert, 1917) | “l’Amérique méridionale” (Houlbert 1917) | Mexico (Lamas 1995) |
| *Eumenia toxea* Godart, 1824 (Lepidoptera: Lycaenidae) | *Eumaeus toxea* (Godart, 1824) | “de l'intérieur de l'Amérique méridionale” [=from the interior of South America] (Godart 1824) | Guatemala, Honduras and Mexico (Faynel & Bálint 2004) |

Taking into consideration the information provided above, it may thus be hypothesised that Boisduval’s “am. m.” on the *Parasa chloris* lectotype is a mislabelling, given that the species and it’s close relatives are identified only from North and Central America.

**References**

Berthold AA. 1827. Latreille’s Naturliche Familien des Thierreichs. Im Verlage des Gr. H.S. priv. Landes-Industrie-Comptoirs.

Boisduval JB, Le Conte JE. 1833. Histoire général et iconographie des lepidoptérès et des chenilles de l’Amérique septentrionale. Roret.

Boisduval JB. 1836. Histoire naturelle des insectes: species général des lépidoptères, Vol. 1. Roret.

d'Orbigny AD. 1835-1847. Voyage dans l'Amérique méridionale: le Brésil, la république orientale de l'Uruguay, la république Argentine, la Patagonie, la république du Chili, la republ. de Bolivia, la républ. du Pérou, Vol. 1–9. Pitois-Levrault.

Druce H. 1887. Lepidoptera-Heterocera. In: Godman FD, Salvin O, editors. Biologia Centrali-Americana; or, contributions to the knowledge of the fauna and flora of Mexico and Central America. Zoology: Insecta, Vol. 1. Taylor & Francis. <https://doi.org/10.5962/bhl.title.730>

Dyar HG. 1891. A revision of the species of *Euclea*, *Parasa* and *Packardia*, with notes on *Adoneta*, *Monoleuca* and *Varina* *ornate* Neum. Trans. Am. Entomol. Soc. 18(2−3):149−158.

Faynel C, Bálint Z. 2004. Supplementary information on neotropical Eumaeini primary type material and further historical specimens deposited in the Muséum national d'Histoire naturelle, Paris (Lycaenidae, Theclinae). Bulletin de la Société entomologique de France 109(3):263–286.

French GH. 1885. Larva of *Parasa chloris*, H.-Sch.  Can. Entomol. 17(9):161−162.

Gerhard B. 1878. Systematisches Verzeichniss der Macrolepidopteren von Nord-Amerika. R. Friedländer & Sohn.

Grote AR, Robinson CT. 1868a. Notes on the North American Lepidoptera in the British Museum and described by Mr. Francis Walker. Trans. Am. Entomol. Soc. 2:67–88.

Grote AR, Robinson CT. 1868b. List of the Lepidoptera of North America. American Entomological Society. <https://doi.org/10.5962/bhl.title.38745>

Grote AR. 1865. Descriptions of North American Lepidoptera - No. 6. Proc. Ent. Soc. Phil. 4:315−330.

Grote AR. 1881. Descriptions of four new species of moths. Papilio 1:4−6.

Grote AR. 1882. New check list of North American moths. [Publisher unknown].

Houlbert C. 1917. Diagnoses de castnies nouvelles et rectification de quelques noms indûment employés. *Etudes de lépidopterologie comparée* 13:49–85, pl.1–4.

Kirby WF. 1892. A synonymic catalogue of Lepidoptera Heterocera (Moths). Vol. 1. Sphinges and Bombyces. Gurney & Jackson. <https://doi.org/10.5962/bhl.title.9152>

Lichy R. 1968. Documentos para servir al estudio de los Sphingidae de Venezuela (Lepidoptera, Heterocera) (12a nota). Boletín de Entomologia Venezolana 18:31–42.

Mann BP. 1879. Descriptions of some Larvae of Lepidoptera, respecting Sphingidae especially. Psyche 2(65−68):265−272.

Morris JG. 1860. Catalogue of the described Lepidoptera of North America. Smithsonian Institution. <https://doi.org/10.5962/bhl.title.27990>

Morton CV. 1973. Studies of fern types, II. *Contr*. U.S. Natl. Herb. 38(6): 215–281.

Packard AS. 1864. Synopsis of the Bombycidae of the United States (1&2). Proc. Ent. Soc. Phil. 3:331−396.

Poiret JLM. 1812. Encyclopédie méthodique. Botanique. Supplément 2. Agasse.

Smith JB. 1891. List of the Lepidoptera of North America. American Entomological Society. <https://doi.org/10.5962/bhl.title.1883>

Stretch HR. 1873. Illustrations of the Zygaenidae & Bombycidae of North America, Vol. 1. [unknown publisher]. <https://doi.org/10.5962/bhl.title.9270>

Wagner DL. 2005. Caterpillars of eastern North America: a guide to identification and natural history. Princeton University Press.
