# Supplementary material for: On the identity of the type species of Parasa (Lepidoptera: Limacodidae): investigations into the Nearctic Parasa chloris and related taxa
Source: Ann Entomol Soc Am. 2025 Jul 14;118(4):276–89. doi: 10.1093/aesa/saaf016 (PMC12285581; doi:10.1093/aesa/saaf016)
Supplement: saaf016_suppl_Supplementary_Materials [file saaf016_suppl_supplementary_materials.zip › saaf016_suppl_Supplementary_Materials_S1.docx]

**S1: Additional material examined**

***Parasa chloris***

**USA.** [No precise locality data] 1 male: ex A.S. Packard, B.M. 1901–134; 2 males: [no label]; 1 male, 1 female: ex H.J. Adams, B.M. 1912–399 (NHMUK). 3 males: North Carolina, near Lake Lure, 4.viii.1951, E.C. Zimmerman, B.M. 1951–481; 1 male: same locality data, 6.viii.1951, B.M. 1951–481; 1 female: New York; 1 male: Pennsylvania; 1 male: New York, Long Island, Barnes Collection; 1 male: Iowa (NHMUK). 1 male: Louisiana, Bossier Parish, Barksdale A.F.B., 32°29′06″N 93°35′30″W, 21.v.1996, D. Adamski, USNMENT01918180, gen. slide no. TT257; 1 male: Virginia, Fairfax County, 1 km E Fairfax City, 38°49.6′N, 77°15.8′W, 11.vii.2008, J. Brown, USNMENT01918176, gen. slide no. TT255; 1 male: Ohio, Summit County, Springfield Township, Springfield Bog Park, 41 00′ 28.5″ N, 081 23′ 56.9″ W, 26.vi.2011, J.D. Hooper, USNMENT01918175; 1 male: Louisiana, Bossier Parish, Barksdale A.F.B., 32°30′45″N 93°32′39″W, 18.v.1996, D. Adamski, USNMENT01918181; 2 specimens (sex unknown): Virginia, Toms Brook, Shenandoah County, 38.923, -78.426, 16.viii.2016, BOLD process IDs: FGCAT386-20, FGCAT355-20; 1 female, Texas, Little Rock Rd., 1.1 mi from FM 326, Big Thicket National Preserve, Hardin County, 30.30528, -94.37153, 23.v.2011, M. Pogue, R. Stone, USNMENT01848904, BOLD process ID: SML487-23; 1 female: Maryland, Tantallon, D.W. Strasburg, 29.vii.1989, USNMENT00945644, BOLD process ID: LNAUT1285-14; 1 male: North Carolina, Balsam, Jackson County, 35.43, -83.09, D.C. Ferguson, 17.vii.1974, USNMENT01237384, BOLD process ID: LNAUV185-16 (USNM); 1 female: Connecticut, Mansfield, 22 Hunter’s Run, Tolland Co., 19.vii.2001, D.L. Wagner, BOLD process ID: LIMA049-05 (University of Connecticut); 1 specimen (sex unknown): Tennessee, Cosby Creek Campground Septic Field, Cocke, 35.76, -83.21, J. Adams, 20.vii.2004, BOLD process ID: LGSM136-04; 1 male: Tennessee, Cosby Creek Campground Septic Field, Cocke, 35.76, -83.21, J. Adams, 20.vii.2004, BOLD process ID: LGSM135-04; 1 specimen (sex unknown): Tennessee, Blount County, BOLD process ID: LGSME118-06 (depository unknown); 1 male: Georgia, Pleasant Valley Hunting Club S of Adairsville, Bartow County, J. Adams, 34.19, -84.52, 07.iii.2006, BOLD process ID: LSEU542-06 (Centre for Biodiversity Genomics).

***Parasa huachuca***

**USA.** [No precise locality data]. 1 female: ex H.J. Turner, B.M. 1961–609 (NHMUK). 1 male: Arizona, Southwestern Research Station, Portal, N31°53′12″ W109°12′28″, 1630 m, 11-20.viii.2012, A. Giusti, BMNH(E) 2012–137; 11 males, Arizona, Santa Rita Mountains, Madera Canyon, 24-25.vii.1971, A. Watson, B.M. 1971–446; 1 female: Pena Blanca Lake, Oro Blanco Mountains, Santa Cruz County, 26.vii.1971, A. Watson, B.M. 1971–446; 2 males: Arizona, Baboquivari Mountains, vii.1925; 1 female: California, ex H.J. Turner, B.M.1961–609 (NHMUK); 1 female: Arizona, Coshise County, Miller Canyon, 24.vii.1991, J. Glaser, USNMENT01978147; 1 male: Arizona, Brown Canyon, Pima County, 31.7724N, 111.5560W, 28.ix.2019, K. Richers, USNMENT01848902; 2 males: Arizona, 1429 Franklin St, Bisbee, Cochise County, 29.vii.2009, A. S. Menke, USNMENT01918177, USNMENT01848901, gen. slide no. TT256, BOLD process IDs: SML485-23, SML484-23; 1 female: Arizona, 5131 Bannock Street, Pueblo Del Sol, Huachuca Mountains, Cochise County, 31.4759, -110.27, 30.viii.1986, R. Wielgus, USNMENT01848900, BOLD process ID: SML483-23 (USNM); 1 female: Arizona, 1.16 km West of Highway 92 on East Miller Canyon Road, 31.427, -110.252, C. Melton, 17.viii.2010, BOLD process ID: CMAZA965-12 (Centre for Biodiversity Genomics).

***Parasa minima***

**USA.** 1 male: Texas, Santa Ana Refuge, Hidalgo County, 31.viii.1986, E.C. Knudson, USNMENT01848905, BOLD process ID: SML488-23; 1 female: Texas, Bentsen Rio-Grande Valley State Park, Hidalgo County, USNMENT01978145; 1 male: Texas, Cameron County (southmost), E.C. Knudson, 27.x.1986, USNMENT01918178, gen. slide no. TT259 (USNM); 1 specimen (sex unknown): Texas, Sabal Palm Audubon Sanctuary, Cameron County, 25.8512, -97.4233, 19.xi.2017, D.L. Wagner, BOLD process ID: WAGL877-18; 1 male: Texas, Hidalgo County, 26.1547, -98.3257, 18.x.2007, C. Bordelon and E.C. Knudson, BOLD process ID: WAGL225-16 (University of Connecticut). **Mexico.** 3 males: 3 mi. E. Galeana, Nuevo Leon, 07.viii.1963, D. Duckworth, USNMENT01848903, USNMENT01848906, USNMENT01848907 BOLD process IDs: SML486-23, SML489-23, SML490-23 (USNM).

***Parasa maysi***

**Belize.** 1 female: Cayo, Chiquibul Forest, Las Cuevas Field Station, 88°59′W, 16°43′N, v.2010, H. Mendel and M.V.L. Barclay, BMNH{E} 2010–54; 1 female: Punta Gorda, x.1914, J.D. Norton, Joicey Collection, B.M. 1925–157; 1 male: Rio Temash, 1934. | J.J. White, B.M.1935–47; 1 male: Las Cuevas, 9-10.xii.1998, N.H. Hall, 16°44.0′N, 88°59.2′W, (NHMUK); **Mexico.** 1 female: Conhuas, Calakmul, Campeche, 18.56, -89.92, 1.xi.2006, E. May, BOLD process ID: LPYPA847-08; 1 female: Reserva Kiuic, Oxkutzcab, Yucatan, 20.089, -89.55, 19.vi.2008, E. Dominguez, BOLD process ID: LYPIE280-09 (El Colegio de la Frontera Sur).

***Parasa cuernavaca***

**Mexico**. 1 male: Zacualpan, vi.1915, Joicey Collection, B.M. 1925–157; 1 male: Zacualpan, vii.1918, Joicey Collection, B.M. 1925–157; 1 male: Zacualpan, Joicey Collection, B.M. 1925–157; 1 male: Zacualpan, ix.1922, Joicey Collection, B.M. 1925–157 (NHMUK), 1 male: Venadio, Sinaloa, 19.ix.1934, gen. slide no. USNM134534 (by Heinrich), USNMENT01848165 (USNM); 2 males: Zacualpan, ix.1915; 1 male: Colima, vi.1924 (ZSM).

***Parasa cf. cuernavaca***

**Costa Rica.** 1 male: Santa Rosa National Park, Guanacaste Province, 11.vi.-2.vii.1985, I.J. Kitching, B.M. 1985–290; 1 male: Candelaria Mountains, 2.viii.1895; C.F. Underwood, Godman-Salvin Collection, B.M. 1898–40; 1 male: Bebedero, C.F. Underwood; 1 male: C.F. Underwood; 1 male: Guapiles (NHMUK); 1 male: Area de Conservacion Guanacaste, 17-SRNP-105661, USNMENT01918183; 1 male: Area de Conservacion Guanacaste, 16-SRNP-104095, USNMENT01918184, gen. slide no. TT258 (USNM); 1 male: Luces, Sector Santa Rosa, Area de Conservacion Guanacaste, Guanacaste, 10.854, -85.609, H. Cambronero and S. Rios, 27.v.2014, BOLD process ID: BLPEG2127-14; 1 male: Sendero Natural, Area de Conservacion Guanacaste, Guanacaste, 10.836, -85.613, 14.xi.2003, Ruth Franco, BOLD process ID: MHMXB413-06; 1 female: Area Administrativa, Sector Santa Rosa, Area de Conservacion Guanacaste, Guanacaste, 10.838, -85.619, G. Pereira, 08.i.2004, BOLD process ID: MHMXB381-06 (University of Pennsylvania); 1 male: Estacion Gongora, Sector Cacao, Area de Conservacion Guanacaste, Guanacaste, 10.884, -85.473, 10.ix.2007, S. Rios and H. Cambronero, BOLD process ID: BLPCE179-08; 1 male: Estacion Gongora, Sector Cacao, Area de Conservacion Guanacaste, Guanacaste, 10.884, -85.473, 11.ix.2007, S. Rios and H. Cambronero, BOLD process ID: BLPCE473-08 (depository unknown); 1 male: Hamburg Farm, Reventazon, F. Nevermann, iii.1928; 1 male: Hamburg Farm, Reventazon, F. Nevermann, iv.1928 (ZSM). **Guatemala.** 1 male: Guatemala City, Rodriguez, Godman-Salvin Collection, B.M. 1898–40. **Mexico.** 1 male: San Jeronimo, Tacaná, Chiapas, 5.x. 1970 (NHMUK).

***Parasa indetermina***

**USA.** [No precise locality data] 3 females, 4 males [No label]; 1 male, 1 female: ex H.J. Adams, B.M. 1912–399; 1 female: ex A.R. Grote, B.M. 1881–116; 1 female: 1920, H.J. Elwes, B.M. 1925–157 (NHMUK). 1 female: H. Fruhstorfer; 1 male: 1891; 2 males, 2 females; 4 males: Texas; 1 male, 1 female: Iowa; 1 male: Virginia; 2 males: Joicey Collection, B.M.1925–157; 3 males, 1 female: Joicey Collection, B.M. 1922–306; 1 male: Pennsylvania, Cornwall, Joicey Collection, B.M. 1922–306; 1 male: Joicey Collection, B.M. 1925–157; 1 female: Texas, Joicey Collection, B.M. 1922–306; 1 female, Pennsylvania, Joicey Collection, B.M. 1925–157, 1 female, Pennsylvania, Joicey Collection, B.M. 1922–306; 1 female, New York, Brooklyn, 18.vi.1902 (NHMUK); 1 male, Maryland, Patuxent Wildlife Research Center, 30.vi.1995, D.C. Ferguson, USNMENT01918182, gen. slide no. TT236 (USNM); 1 male: Cape May, New Jersey, 38.94, -74.97, 19.vi.1905, M.E. Epstein, BOLD process ID: LTOLB893-11 (University of Maryland); 1 female: Georgia, Taylor’s Ridge, Walker County, 34.42, -85.1, 02.vii.2006, J. Adams and I. Finkelstein, 06-JKA-0554, BOLD process ID: LSEU554-06 (Centre for Biodiversity Genomics).
